# Unequal distribution of health human resource in mainland China: what are the determinants from a comprehensive perspective?

**DOI:** 10.1186/s12939-018-0742-z

**Published:** 2018-02-27

**Authors:** Dan Li, Zhongliang Zhou, Yafei Si, Yongjian Xu, Chi Shen, Yiyang Wang, Xiao Wang

**Affiliations:** 1 School of Public Policy and Administration, Xi’an Jiaotong University, Xi’an, Shaanxi 710049 People’s Republic of China; 2 Global Health Institute, Xi’an Jiaotong University Health Science Center, Xi’an, People’s Republic of China; 3 International Business School Suzhou, Xi’an Jiaotong-Liverpool University, Suzhou, People’s Republic of China

**Keywords:** Inequality, Health human resource, Concentration index, Decomposition analysis

## Abstract

**Background:**

The inequality of health human resource is a worldwide problem, and solving it also is one of the major goals of China’s recent health system reform. Yet there is a huge disparity among cities in mainland China. The aim of this study is to analyze the distribution inequality of the health human resource in 322 prefecture-level cities of mainland China in 2014, and to reveal the facets and causes of the inequalities.

**Methods:**

The data for this study were acquired from the provincial and municipal Health Statistics Yearbook (2014) and Statistical Yearbook (2014), the municipal National Economic Bulletin (2014), and the official websites of municipal governments, involving 322 prefecture-level cities. Meanwhile, Concentration Index was used to measure the magnitude of the unequal distribution of health human resource. A decomposition analysis was employed to quantify the contribution of each determinant to the total inequality.

**Results:**

The overall concentration index of doctors and nurses in mainland China in 2014 was 0.1038 (95% CI = 0.0208, 0.1865) and 0.0785 (95% CI =0.0018, 0.1561). Decomposition of the concentration index revealed that economic status was the primary contributor (58.5% and 57%) to the inequality of doctors and nurses, followed by the Southwest China (19.1% and 18.6%), urbanization level (− 13.1% and − 12.8%), and revenue (8.0% and 7.8%). Party secretaries with Master degree (7.0%, 6.8%), mayors who were 60 years old or above (6.3%, 6.1%) also were proved to be a major contributor to the inequality of health human resource.

**Conclusions:**

There was inequality of health human resource distribution which was pro-rich in mainland China in 2014. Economic status of the cities accounted for most of the existing inequality, followed by the Southwest China, urbanization level, revenue, party secretaries with Master degree, and mayors who were 60 years old or above in respective importance. Besides, the party secretaries and mayors also had certain influence on the allocation of health human resource. The tough issue of HHR inequality should be addressed by comprehensive measures from a multidisciplinary perspective.

## Background

The situation of “Kan Bing Nan” (adequate medical treatment was difficult to access) is one of the serious reflections of unequal distribution of health human resource (HHR) in mainland China. Although the equality of HHR allocation is highly ranked in the policy agenda, many people still are plagued by HHR inequalities indeed.

An equal allocation of HHR, implying most people are able to equally access HHR when it is needed [1, 2], has a profound impact on human health, as well as efficiency, healthy and sustainable development of health services [3]. Thus, it is rather necessary to study the inequality of HHR and the determinants, which in turn can also be referential for assessing the effects of the health system reform in mainland China.

The inequality of HHR can be determined by numerous factors, such as economic development which has an impact on the allocation of HHR within and among countries [4–8], the geographic size of areas [9, 10], and the level of urbanization in China, implying that areas with a better socioeconomic environment would attract more HHR [11, 12]. Moreover, as population density has been identified as a factor contributing to the inequality of HHR [13], empirical results indicated that HHR in the southeast of China was redundant whereas insufficient in the west [14–16]. In addition, fiscal revenue and ratio of health expenditure to fiscal expenditure naturally should be included, since once they are fixed, the equal HHR distribution will be guaranteed [17, 18].

In addition, notwithstanding one of the most prevalent concepts of government official promotion called “promotion tournament” encourages local officials to compete against each other based on a series of performance evaluation indicators [19], the assessment of public resources indeed has a positive effect on the promotion of local officials according to China’s scientific assessment system, of which the effect is more notable in big cities, in particular the cites with strong administrative force [20]. Given the decisive role of the party secretaries and mayors in municipal government in mainland China, it is reasonable to take the details of party secretaries and mayors into consideration for the study of public resource allocation [21], including local health resources.

Differ from most prior studies based on province-level data, using the data of 322 prefecture-level cities in mainland China, this study introduces a decomposition analysis into the research of HHR inequalities. Furthermore, as observers have long suspected the impact of the substantive individuals behind the government’s actions--officials, we offer a tentative explanation for the HHR inequality in terms of party secretaries and mayors from both macro and micro perspectives. By examining the inequality of HHR distribution in mainland China and decomposing the measured inequality into determinants, this study reveals the contribution of each determinant to the inequality, which may be referential for promoting reasonable HHR distribution in China.

## Methods

### Data

HHR involves “all people engaged in actions whose primary intent is to enhance health” [22], such as licensed (assistant) doctors and registered nurses, pharmacists, technicians, and other technical staff. Data of the licensed (assistant) doctors and registered nurses were deployed in this study.

The data on HHR were obtained from the provincial and municipal Health Statistics Yearbooks (2014), and socio-economic data were obtained from the provincial and municipal Statistics Yearbooks (2014) and municipal National Economic Bulletin (2014). The data include number of doctors and nurses, Gross Domestic Product (GDP), population density, urbanization level, fiscal revenue and ratio of health expenditure to fiscal expenditure, region, based on which the values of indexes for this study were calculated (e.g. the number of doctors per 10,000 people, the number of nurses per 10,000 people and Per capita GDP). These data were released by the Chinese government that are reliable.

The HHR allocation has the characteristics of time delay and time accumulation [23]. However, under the fiscal decentralization system and the appointment system in China, as to demonstrate personal strength, projects related to economic growth are taken into account precedingly rather than emphasizing on public resources [24–26]. So we chose the data of party secretaries and mayors at the municipal level in 2012, 2 years ahead of 2014. The details of party secretaries and mayors were also deployed for this study, including age, length of service, sex, nation and education. The data were obtained from the official website of municipal government. Regarding the tenure of many party secretaries and mayors may be less than one calendar year, the data of party secretaries and mayors were selected by the rules as follows:

- If there is no replacement of the party secretary (mayors) in 2012, his or her data will be collected.
- If there is a replacement of party secretary (mayor) in 2012, the data of the party secretary and mayor serving more than 6 months in the city will be collected.
- If there are many replacements of party secretary (mayor) in 2012, and no party secretary (mayor) has served for more than 6 months, then the data of the one serving with the longest term in the city will be collected.

**Fig. 1:**
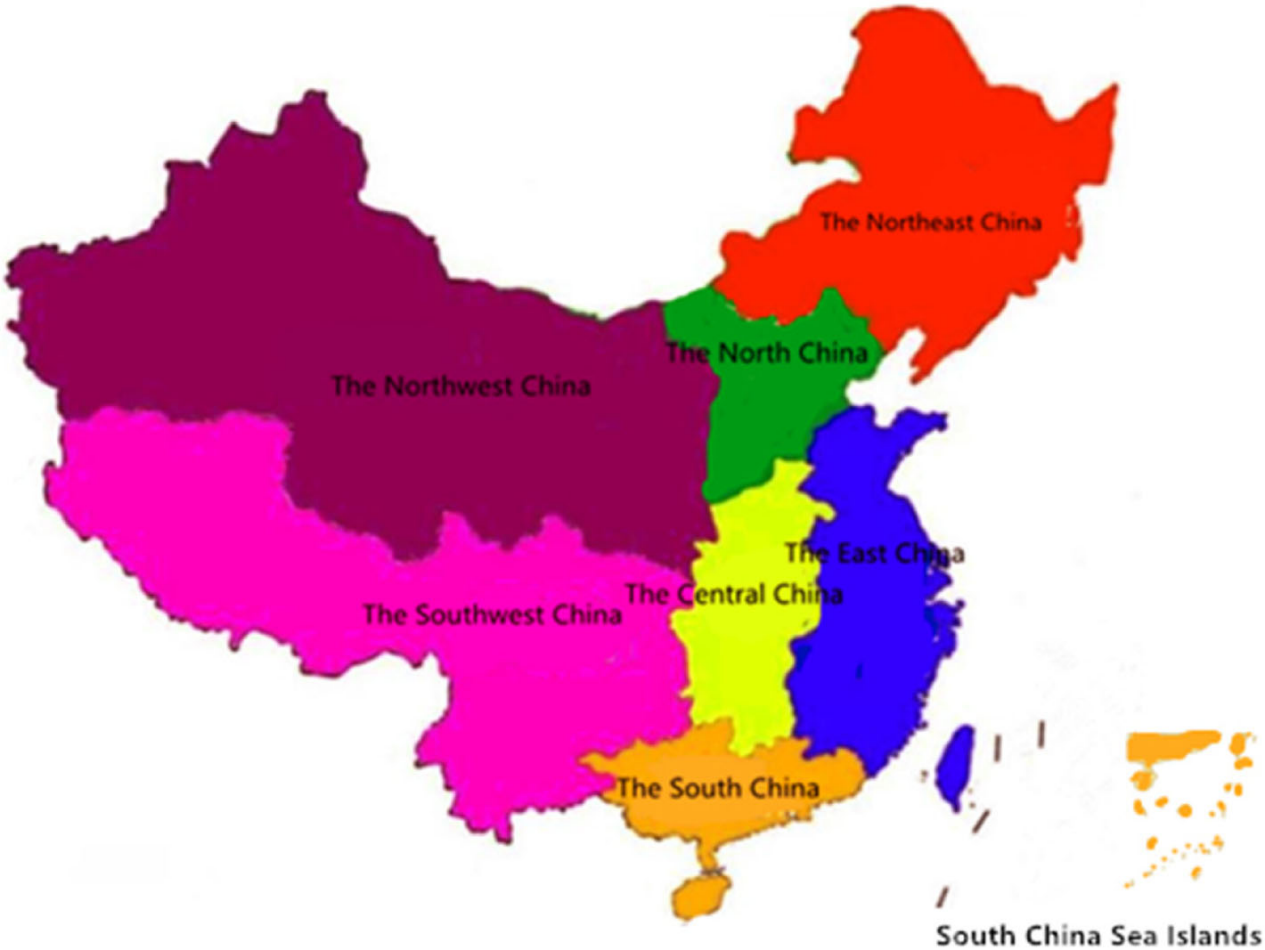
Regional distribution in China. It showed seven regional distribution in China in 2014

As shown in Fig. 1, mainland China consists of 322 prefecture-level cities, and is usually divided into seven geographical regions according to geographic location, population, environment, and other factors: the East China, the South China, the North China, the Central China, the Southwest China, the Northwest China, and the Northeast China. In this study, OLS linear regression model [27, 28] were deployed to examine the association of several variables with the number of HHR per 10,000 people. Population density, urbanization level, fiscal revenue and ratio of health expenditure to fiscal expenditure, Per capita GDP, the details of party secretaries and mayors (age, length of service, sex, nation and education) and region were involved as the independent variables, and the dependent variables were the number of doctors per 10,000 people and the number of nurses per 10,000 people.

### Measuring inequality

Concentration Curve (CC) and Concentration Index (CI) have been widely used to depict the inequality of HHR distribution [29–31]. The CC gives a complete picture of shares of the health variable (y-axis) according to the economic status variable (x-axis). In the situation of completely equal distribution of HHR, regardless of economic status, the CC will be a 45-degree line called the “line of equality”. If the health variable has higher (lower) values among poorer people, the CC would lay over (under) the line of equality and vice versa.

The CI is bound between − 1 and 1, and it is zero if there is no income-related inequality of HHR. If it has a positive (negative) value, there will be a pro-rich (pro-poor) inequality in HHR. The CI is calculated using Eq. 1 [32]:

1 $$ C=\frac{2}{\mu}\operatorname{cov}\left(\mathrm{h},\mathrm{r}\right) $$

Where C is CI, *h* is HHR, μ is the mean of health human resource, r is the fractional rank of income, ranging from 0 to 1. The rank of the i individual is: *r*_*i*_ = *i*/*N* in which N is the number of individuals.

In most cases, health outcome variables are seldom continuous. Marginal effect can be opted to approximate the decomposition analysis [33]. A linear approximation of the non-linear estimation can be presented with Eq. 2:

2 $$ {y}_i={\alpha}^m+\sum \limits_jk{\beta_k}^m{x}_{ki}+{\mu}_i $$

Where *β*_*k*_^*m*^ is the marginal effects (dy/dx) of each x; *μ*_*i*_ indicates the error term generated by the linear approximation. The concentration index for the interested variable can be obtained with Eq. 3 [34]:

3 $$ C=\sum \limits_j\left({\beta}_j^m\overline{x}/\mu \right){C}_j+\sum \limits_k\left({\gamma}_k^m\overline{z}/\mu \right){C}_k+{GC}_u/\mu $$

## Results

The characteristics of cities were described in Table 1, including the mean values and standard deviation of population density, geographical size, population size, Per GDP, revenue, proportion of health expenditure on fiscal expenditure, urbanization, and the details of party secretary and mayors. Most party secretaries were at age of 54 or above (60.53%), serving for less than 30 years (39.18%); the majority of party secretaries were male (95%), Hans (91.47%), and M. A/MSc (54.12%). Meanwhile, most mayors were categorized into the age group of 54 years old or below (84.5%), and the service-length group of 30 years and below (61.4%); the majority of party secretaries were male (92.94%), Hans (82.06%), and M. A/MSc (42.11%).

**Table 1:** Characteristics of 322 prefecture-level cities

Characteristics	All (*N* = 322)	
	Mean (SD)	
Population density(person/km^2^)	166.36 ± 95.52	
Geographical size (/km^2^)	29,632.74 ± 57,234.56	
Population size (/ten thousand people)	386.4 ± 346.16	
Pergdp (/yuan)	46,836.71 ± 10,628.22	
Revenue (/million yuan)	887,822.1 ± 2,738,395	
Proportion of health expenditure on fiscal expenditure(%)	9.58 ± 2.79	
Urbanization (%)	51.29 ± 17.16	
(Characteristics of Party Secretary and Mayor)	Party Secretary N(%)	Mayor N(%)
Age		
<=54	207(60.53)	289(84.5)
55–59	125(36.55)	48(14.04)
> = 60	10(2.92)	5(1.46)
Length of service Secretary		
<=30	134(39.18)	210(61.4)
31–37	130(38.01)	100(29.24)
> = 38	78(22.81)	32(9.36)
Sex		
Men	323(95)	316(92.94)
Women	17(5)	24(7.06)
Nation		
Han	311(91.47)	279(82.06)
Minority nationality	29(8.53)	61(17.94)
Education		
College and below	13(3.82)	32(9.35)
Bachelor	76(22.35)	84(24.56)
Master	184(54.12)	144(42.11)
PhD.	67(19.71)	79(23.10)

Table 2 presents the adjusted associations between HHR inequality and its determinants. It was found that per GDP, revenue and the age group of 55–59 of party secretaries increased the odds of doctors’ inequality, whereas other factors, such as the Central China, the Southwest China and the Northwest China decreased the odds of doctors’ inequality. Simultaneously, per GDP, revenue, and the age group of 55–59 of party secretaries increased the probability of nurses’ inequality, whereas other factors, such as population size, the age group of > = 60 of mayors, the Central China, the Southwest China and the Northwest China decreased the probability of nurses’ inequality.

**Table 2:** Association between the number of HHR per 10,000 people and the determinants

Variable	Doctor		Nurse	
	dy/dx	Std. Err.	dy/dx	Std. Err.
Population density	0.0047	0.0059	0.0007	0.0072
Geographical size	0.0000	0.0000	0.0000	0.0000
Population size	−0.0019	0.0021	−0.0053*	0.0025
Per GDP	0.0001***	0.0000	0.0001**	0.0000
Revenue	0.0000**	0.0000	0.0000***	0.0000
Proportion of health expenditure on fiscal expenditure	−0.3165	0.2349	− 0.0795	0.2857
Urbanization	−0.0530	0.0525	0.0447	0.0647
Age of Party Secretary				
<=54	Ref		Ref	
55–59	3.0421*	1.5213	4.0075*	1.8490
> = 60	4.5746	4.2659	9.0063	5.1814
Length of service of Party Secretary				
<=30	Ref		Ref	
31–37	−2.8585	1.3100	−0.4746	1.5912
> = 38	−2.9771	1.9021	−2.7556	2.3118
Sex of Party Secretary				
Men	Ref		Ref	
Women	−0.1618	2.1345	−1.2070	2.5917
Nation of Party Secretary				
Han	Ref		Ref	
Minority nationality	−0.9821	2.3729	−0.5436	2.8808
Education of Party Secretary				
College and below	Ref		Ref	
Bachelor	−4.2614	2.9432	−0.2802	3.5753
Master	−4.7723	2.8285	−0.6080	3.4364
PhD.	−1.9647	3.0257	0.7101	3.6741
Age of Mayor				
<=54	Ref		Ref	
55–59	−0.7691	1.9983	1.4672	2.4261
> = 60	−14.6293	12.2678	−32.7915*	14.8965
Length of service of Mayor				
<=30	Ref		Ref	
31–37	2.5028	1.3881	3.1671	1.6852
> = 38	1.6079	2.5343	−1.3347	3.0778
Sex of Mayor				
Men	Ref		Ref	
Women	−2.0835	2.1757	−2.7475	2.6415
Nation of Mayor				
Han	Ref		Ref	
Minority nationality	3.1111	2.2851	0.6080	2.7743
Education of Mayor				
College and below	Ref		Ref	
Bachelor	−3.7471	2.0431	−3.0142	2.4809
Master	−5.6737	8.1371	−9.1420	9.8788
PhD.	−1.5799	1.0905	−1.2583	1.3251
Region				
The East China	Ref		Ref	
The North China	−2.5839	2.0042	−7.9930	2.4339
The Central China	−3.3095*	1.6322	−3.9606*	1.9827
The South China	−3.8081	2.0588	−1.8814	2.4997
The Southwest China	−7.0356***	1.8410	−11.0550***	2.2371
The Northwest China	−2.8289*	2.4728	−15.3235***	3.1233
The Northeast China	−3.7785	1.9349	−6.1465	2.3506

The Symbol of “*” is defined by a *P* value < 0.05; the Symbol of “**” is defined by a P value < 0.01; the Symbol of “***” is defined by a P value < 0.001

**Fig. 2:**
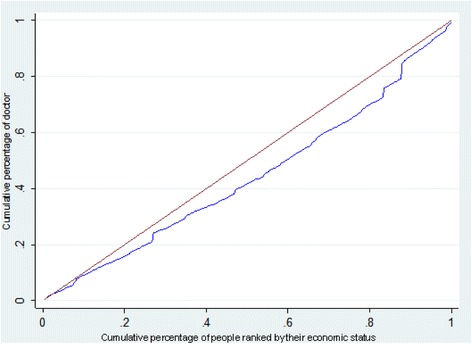
Concentration curve of doctors in China, 2014. The brown line, running from the lower left corner to the upper right corner, is the equality line. The blue line below the equality line represents the concentration curve

**Fig. 3:**
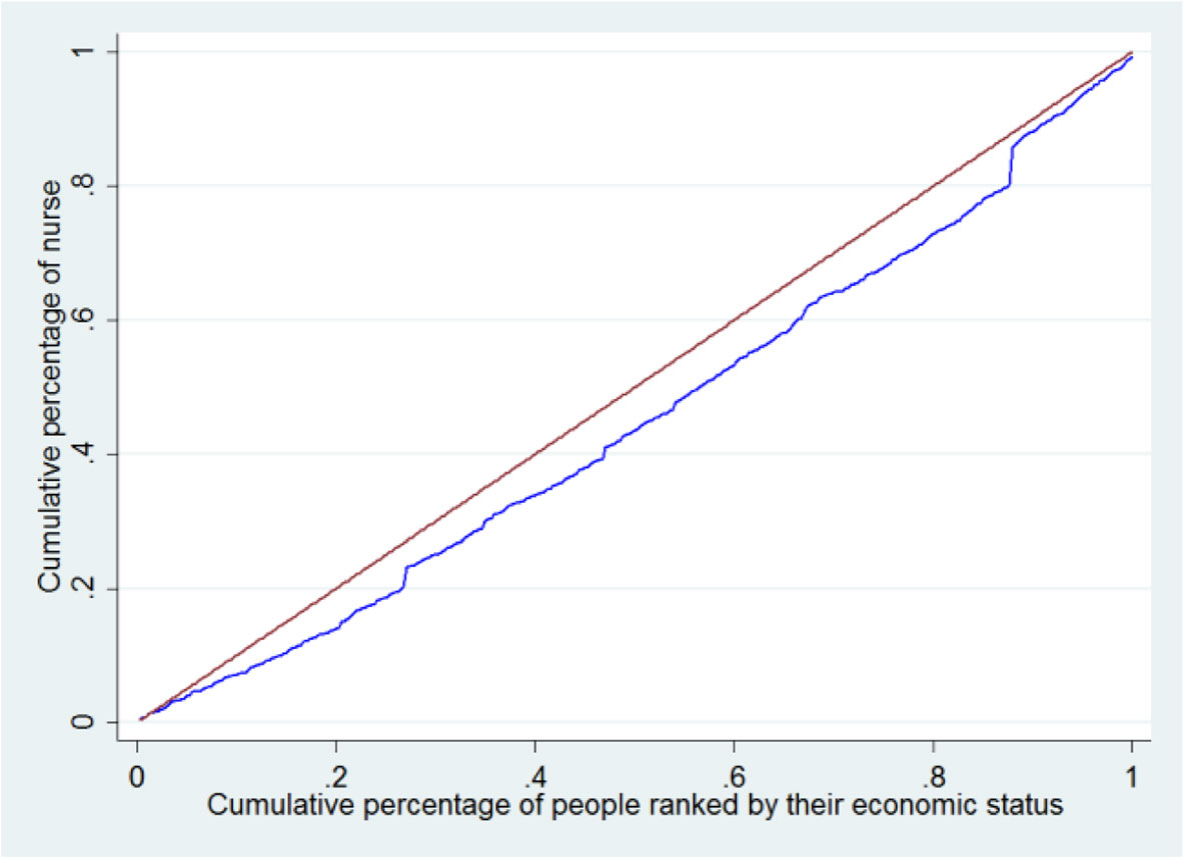
Concentration curve of nurses in China, 2014. The brown line, running from the lower left corner to the upper right corner, is the equality line. The blue line below the equality line represents the concentration curve

The elementary concentration index and concentration curve of the doctors were depicted in Fig. 2. The concentration curve lays below the 45-degree line (the line of equality), and the corresponding elementary concentration index is 0.10381 (95% CI = 0.0208, 0.1865), indicating that doctors are more concentrated in the cities with economic advantages favoring the rich. Figure 3 illustrates the CC of nurses, indicating that cities with less economic advantages do suffer from higher inequality than those with more economic advantages. The overall CI of the inequality of HHR in China is 0.0785 (95% CI =0.0018, 0.1561), again revealing that doctors and nurses are more concentrated in the cities with economic advantages favoring the rich.

The elasticities, CIs of each exploratory variable, absolute contributions to C, and percentage contributions to C, are presented in Table 3. In this table, C_k_ represents the CIs of the explanatory variables in column 3. In terms of doctors, positive values of the variables such as per GDP, revenue, and urbanization, indicate that they are more concentrated in the cities with more economic advantages and vice versa. Positive values in regard to nurses show the similar results. Additionally, the absolute contributions and the percentage of contributions of each variable to the observed inequality are presented in column 4 and column 5. Decomposition analysis indicates that the economic status accounts for the largest proportion in the inequality of doctors (58.5%), the Southwest China (19.1%), urbanization level (− 13.1%), revenue (8%), Party secretaries with master degree (7.0%) and age group of mayors 60 years old or above (6.3%) following respective importance. Moreover, the economic status makes the largest contribution to the inequality of nurses (57%), urbanization level (− 12.8%), the Southwest China (18.6%), revenue (7.8%), Party secretaries with master degree (6.8%) and age group of mayors 60 years old or above (6.1%) following respective importance. The component analysis shows that variables in the current model can explain approximately 81.8% of the inequality in doctors and approximately 77.4% of the inequality in nurses.

**Table 3:** Decomposition analysis of contribution index of HHR in China

Variable	Doctor				Nurse			
	Elasticity	C_k_	Absolute contribution to C	Percentage contribution to C	Elasticity	C_k_	Absolute contribution to C	Percentage contribution to C
Population density	0.0298	0.0143	0.0004	0.5	0.0291	0.0143	0.0004	0.5
Geographical area	− 0.0211	− 0.0985	0.0021	2.6	− 0.0206	− 0.0985	0.0020	2.6
Population size	−0.0283	0.0337	− 0.0010	− 1.2	− 0.0276	0.0337	− 0.0009	− 1.2
Pergdp	0.1384	0.3316	0.0459	58.5	0.1349	0.3316	0.0447	57.0
Revenue	0.0305	0.2074	0.0063	8.0	0.0297	0.2074	0.0062	7.8
Proportion of health on fiscal expenditure	−0.1146	− 0.0444	0.0051	6.5	−0.1117	− 0.0444	0.0050	6.3
Urbanization	−0.1027	0.1003	−0.0103	−13.1	− 0.1001	0.1003	− 0.0100	− 12.8
Age of Party Secretary								
<=54	Ref			1.0	Ref			
55–59	0.0420	0.0190	0.0008	0.7	0.0410	0.0190	0.0008	0.99
> = 60	0.0051	0.1139	0.0006	0.0	0.0049	0.1139	0.0006	0.72
Length of service of Party Secretary								
<=30	Ref				Ref			
31–37	−0.0411	0.0059	−0.0002	− 0.3	− 0.0400	0.0059	− 0.0002	− 0.3
> = 38	− 0.0257	0.0629	− 0.0016	− 2.1	− 0.0250	0.0629	− 0.0016	− 2.0
Sex of Party Secretary								
Men	Ref				Ref			
Women	−0.0003	0.2182	−0.0001	− 0.1	−0.0003	0.2182	−0.0001	− 0.1
Nation of Party Secretary								
Han	Ref				Ref			
Minority nationality	−0.0032	−0.1237	0.0004	0.5	−0.0031	−0.1237	0.0004	0.5
Education of Party Secretary								
College and below	Ref				Ref			
Bachelor	−0.0360	0.0243	− 0.0009	−1.1	−0.0351	0.0243	−0.0009	−1.1
Master	−0.0976	−0.0560	0.0055	7.0	−0.0952	−0.0560	0.0053	6.8
PhD.	−0.0146	0.0924	−0.0014	−1.7	−0.0143	0.0924	−0.0013	−1.7
Age of Mayor								
<=54	Ref				Ref			
55–59	−0.0041	0.2041	−0.0008	−1.1	− 0.0040	0.2041	− 0.0008	−1.0
> = 60	−0.0081	− 0.6071	0.0049	6.3	−0.0079	− 0.6071	0.0048	6.1
Length of service of mayor								
<=30	Ref				Ref			
31–37	0.0277	0.0021	0.0001	0.1	0.0270	0.0021	0.0001	0.1
> = 38	0.0057	0.2531	0.0014	1.8	0.0055	0.2531	0.0014	1.8
Sex of Mayor								
Men	Ref				Ref			
Women	−0.0056	0.0833	−0.0005	−0.6	−0.0054	0.0833	−0.0005	− 0.6
Nation of Mayor								
Han	Ref				Ref			
Minority nationality	0.0211	−0.1972	−0.0042	−5.3	0.0206	−0.1972	−0.0041	−5.2
Education of Mayor								
College and below	Ref				Ref			
Bachelor	−0.0125	0.1662	−0.0021	−2.6	− 0.0121	0.1662	− 0.0020	−2.6
Master	−0.0006	− 0.2963	0.0002	0.2	−0.0006	− 0.2963	0.0002	0.2
PhD.	−0.0252	0.0085	−0.0002	−0.3	− 0.0246	0.0085	− 0.0002	−0.3
Region								
The East China	Ref				Ref			
The North China	−0.0091	0.4007	−0.0036	−4.6	− 0.0088	0.4007	− 0.0035	−4.5
The Central China	−0.0169	− 0.0213	0.0004	0.5	−0.0164	− 0.0213	0.0003	0.4
The South China	−0.0159	0.0209	−0.0003	−0.4	− 0.0155	0.0209	− 0.0003	−0.4
The Southwest China	−0.0430	−0.3478	0.0150	19.1	−0.0419	−0.3478	0.0146	18.6
The Northwest China	−0.0157	−0.1551	0.0024	3.1	−0.0153	−0.1551	0.0024	3.0
The Northeast China	−0.0171	0.1100	−0.0019	−2.4	−0.0167	0.1100	−0.0018	−2.3
Total				81.8				77.4

## Discussion

This study explored the association between a variety of socio-demographic variables and the number of HHR per 10,000 people. In line with most prior studies, our study found that the increase of GDP and revenue were positively associated with the number of HHR per 10,000 people. Differ from a few studies suggesting insignificant association between the age of officials and the number of HHR, we found that age was positively associated with the number of HHR per 10,000 people in the age group of 55–59 years old. The “age ceiling” of the party secretaries and mayors in China could partially explain that: when a party secretary or mayor is more than 55 years old, he or she is less likely to be promoted, and would be less keen on the GDP growth that is a key indicator of his or her work performance. Conversely, he or she will be more likely to undertake more responsibilities for the provision of public services such as health services. Our findings showed that population size was negatively associated with the number of HHR per 10,000 people. This might be partly owing to the fixed standards of nursing number, which is based on the bed nursing ratio. Thus, as the population grows, the nurses per 10,000 people is reduced. Geographically, cities in the Central China, the Southwest China and the Northwest China were associated with decreasing numbers of HHR per 10,000 people. That may be due to the advantages of the economically developed regions assembling the HHR in economically undeveloped regions.

Consistent with most previous province-level studies conducted in mainland China [35–37], our study found that HHR inequality still existed in China, and economy and region took up substantial proportions in the total HHR inequality. However, a small number of studies focused on the HHR inequality at the municipal level. As expected, this paper reveals the huge discrepancy in HHR allocation among different cities in mainland China. Furthermore, the CIs of doctors and nurses indicate that the HHR distribution in mainland China favors the cities with higher levels of economic development, and the inequality of nurses is even worse than the doctors. It highlights the necessity of optimizing the HHR structure, especially the nursing staff team, to reduce the total HHR inequality.

Revealing the HHR inequality and its determinants [38, 39], our decomposition analysis corresponds to the results of prior studies conducted in mainland China [40]—the economic status explains most of the existing HHR inequality. And there is growing evidence that economic development has a significant impact on Chinese health input [41]. This may be due to the increasing demand for health services, and more doctors and nurses are willing to work in economy-developed cities which can provide better salaries, benefits, working conditions, and opportunities for development. Nevertheless, how to balance the flow of HHR among cities with different economic conditions is still one of the key issues to be addressed for the health reform.

Another explanatory variable with relatively large contributions to the HHR inequality is urbanization level. The policies of urbanization and the Urban-Rural Dual System in mainland China can explain the institutional factors causing the widening urban-rural inequality of HHR. Indeed, the government need to formulate appropriate strategies to tackle the existing urban-rural inequalities of HHR and realize well-balanced development of HHR between urban rural areas.

In addition, the disparity of local government revenue is another contributor to the HHR inequality. The coexistence of highly centralized political power and the highly decentralized economic power is the unique institutional arrangements since the reform and opening-up of China [42]. Furthermore, coupled with Fiscal Decentralization Policy in mainland China, economic development has generally boosted government investment in HHR, resulting in comparatively more fiscal expenditure on the HHR flowed into cities with better economic status.

In line with prior studies [43–45], the regional inequality also makes a substantial contribution to the HHR inequality in China. The reform and open-up policy in China is a significant driving force towards regional divergence, and the regions with a better socioeconomic environment could assemble more HHR, leading to the regional HHR inequality. The policy on boosting private medical institutions in 2009 [46] can also give an explanation for the HHR inequality, which pushes the doctor and nurses who are more enthusiastic moving into the regions with a better socioeconomic environment. Nevertheless, the role played by regional preferential policies cannot be ignored. The Southwest China, for example, favored by regional preferential policies, such as “Development of the Western Region in mainland China” introduced in 2000 [47], has better HHR allocation. To reduce regional inequality, a well-functioning regional layout, such as setting up the communication and cooperation mechanism of HHR in different regions is needed to improving the existing HHR allocation.

Many studies [48, 49] ware conducted in terms of the promotion incentives for local officials, which revealed better educational background played an important role in the promotion of officials. For a long time, Chinese governments implement the “promotion tournament system” based on economic growth assessment. For the party secretaries who are more likely to be promoted, they will likely pay more attention to economic growth. Pursuing moderate economic growth can improve social undertakings, but excessive economic growth may impair the performance of other social dimensions, including the HHR distribution [50, 51]. Interestingly, our study also finds that age is negatively associated with the HHR inequality only if the mayors are 60 years old or above, supported by some published articles [48, 52–54]. According to China’s retirement policy, the general retirement age of bureau-level officials is 60 years old. However, the officials who are on a higher level will be relegated from a leading post after 60 years old, instead of retiring. Therefore, officials who are over 60 years old cannot have sufficient power to vigorously develop public health and medical services.

Last but not least, this study also some limitations for further research. First, some prefecture-level governments cannot completely control the counties subordinate to them, since the government of the counties have independent decision-making power on matters within their administrative areas. Future studies thus may seek to investigate on the county level. Another limitation is the lack of access to comprehensive data. A longitudinal comparative analysis will be useful to reveal the tendency of HHR inequalities over time, as well as the causes.

## Conclusions

Our study revealed the facets and causes of the HHR inequalities in 322 prefecture-level cities in mainland China in 2014. It also provided important insights into the HHR inequality with regard to party secretaries and mayors. Pro-rich inequality of health human resource distribution was observed. The cities with lower economic status did suffer from higher HHR inequality than those with higher economic status. Most factors, such as economic status, Southwest China, revenue, party secretaries with master degree, and mayors who were 60 years old or above contributed to increasing the degree of pro-rich inequality. This pro-rich inequality was partially offset by urbanization level. It is crucial to conduct a comprehensive analysis to understand the inequality in health human resource allocation and develop comprehensive measures to address this issue from a multidisciplinary perspective.

## Abbreviations

CPC: Communist Party of China
GDP: Gross domestic product
HHR: Health human resources
WHO: World Health Organization
